# Detection of PCV2d in Vaccinated Pigs in Colombia and Prediction of Vaccine T Cell Epitope Coverage against Circulating Strains Using EpiCC Analysis

**DOI:** 10.3390/vaccines12101119

**Published:** 2024-09-29

**Authors:** Diana S. Vargas-Bermudez, Alixs Constanza Gil-Silva, María F. Naranjo-Ortíz, José Darío Mogollón, Jair F. Gómez-Betancur, José F. Estrada, Álvaro Aldaz, Harold Garzón-González, José Angulo, Dennis Foss, Andres H. Gutierrez, Jairo Jaime

**Affiliations:** 1 Universidad Nacional de Colombia, Sede Bogotá, Facultad de Medicina Veterinaria y de Zootecnia, Centro de Investigación en Infectología e Inmunología Veterinaria (CI3V), Bogotá 111321, Colombia; dsvargasb@unal.edu.co (D.S.V.-B.); josedmogollon@yahoo.es (J.D.M.); 2 Zoetis de Colombia, Titan Plaza Centro Empresarial, Bogotá 111021, Colombia; alixs.gil@zoetis.com (A.C.G.-S.); jairfernando.gomez@zoetis.com (J.F.G.-B.); josefernandoestrada.pineda@gmail.com (J.F.E.); 3 Zoetis Inc., 10 Sylvan Way, Parsippany, NJ 07054, USA; alvaro.aldaz@zoetis.com; 4 Grupo de Investigación en Ciencias Biomédicas (GICBUPTC), Universidad Pedagógica y Tecnológica de Colombia (UPTC), Tunja 150003, BOY, Colombia; harold.garzon@uptc.edu.co; 5 Zoetis Inc., 1040 Swabia Ct, Durham, NC 27703, USA; jose.angulo@zoetis.com; 6 Zoetis Inc., 333 Portage St, Kalamazoo, MI 49007, USA; dennis.l.foss@zoetis.com; 7 EpiVax Inc., Providence, RI 02909, USA; agutierrez@epivax.com

**Keywords:** porcine circovirus type 2 (PCV2), PCV2d, porcine-circovirus-associated diseases (PCVADs), cell-mediated immunity (CMI), immunoinformatic tools, EpiCC analysis

## Abstract

Porcine circovirus type 2 (PCV2) is strongly linked to a group of syndromes referred to as porcine-circovirus-associated diseases (PCVADs), which are controlled through vaccination; however, this does not induce sterilizing immunity but is instead involved in the evolution of the virus and is considered a factor in vaccine failure. This study sampled 84 herds (167 pigs) vaccinated against PCV2 and with clinical signs of PCVADs in five provinces across Colombia. PCV2 was identified and further characterized at the molecular level via genotyping and phylogenetic reconstructions. In addition, PCV2-associated lesions were examined via histopathology. Furthermore, the PCV2-Cap sequences retrieved were compared with three vaccines via the EpiCC tool and T cell epitope coverage. The prevalence of PCV2 was 82% in pigs and 92.9% in herds. The highest viral loads were identified in lymphoid tissue, and PCV2d emerged as the most predominant in pigs and herds (93.4% and 92.3%). Sequences for PCV2-ORF2 (*n* = 57; 55 PCV2d and 2 PCV2a) were determined, and PCV2d sequences were highly similar. The most common pneumonia pattern was suppurative bronchopneumonia, while the most common lung lesion was exudation in the airways; in lymphoid tissue, there was lymphoid depletion. The bivalent vaccine (PCV2a and PCVb) exhibited a higher EpiCC score (8.36) and T cell epitope coverage (80.6%) than monovalent PCV2a vaccines. In conclusion, PCV2d currently circulates widely in Colombia. Despite vaccination, there are clinical cases of PCV2, and immunoinformatic analyses demonstrate that bivalent vaccines improved the average coverage.

## 1. Introduction

Circoviruses are classified under the *Circoviridae* family, genus Circovirus [1]. The swine population is affected by four porcine circoviruses (PCV), including PCV1, PCV2, and the novel PCV3 and PCV4 [2,3]. Structurally, PCVs are small non-enveloped viruses that have circular single-stranded DNA (cssDNA) as their genome with a length between 1.7 and 2 kb [4]. Specifically, the genome of PCV2 has at least ten open reading frames (ORFs), the main three being ORF1, ORF2, and ORF3 [5]. ORF1 encodes for the replicase protein, which participates in viral replication [6], while ORF2 encodes for the structural protein capsid (Cap), which is the most immunogenic [7]. Additionally, ORF3 encodes for an apoptosis-inducing protein that contributes to viral pathogenesis [8]. PCV2 was discovered in Canada in 1998, where it was associated with post-weaning multisystemic wasting syndrome (PMWS) [9]; this clinical manifestation was the most common for several years and was responsible for high economic losses in the swine industry worldwide [10]. Evolutionarily, PCV2 has been changing and, to date, eight genotypes (PCV2a through PCV2h) have been identified [11]. Importantly, these lack a similar global geographical distribution and not all induce severe clinical signs in pigs [12]. PCV2a, PCV2b, and PCV2d are considered the primary genotypes due to their high prevalence, with PCV2d being the predominant genotype worldwide today [13]. The evolutionary capacity of PCV2 has been explained by two reasons: (i) PCV2 exhibited the highest nucleotide (nt) substitution rate (1.2 × 10^−3^ substitutions/site/year) among the identified ssDNA viruses [13] and (ii) the vaccination pressure has been suggested as a contributing factor through the selection of immune escape variants [14]. Prior phyloevolution investigations of PCV2 have demonstrated that the main genotypes have arisen in periodic waves, where they are established in herds, then increasing their prevalence among swine populations, spreading through trade routes, and finally being replaced by new emerging genotypes [15]. In Colombia, the information about the diversity of PCV2 needs to be improved, where prior investigations in 2014 reported the circulation of PCV2b in 82% and 18% for PCV2a [16], while, since 2017, PCV2d has been identified as the predominant genotype [17].

PCV2 induces pathological conditions in pigs, grouped and classified under porcine-circovirus-associated diseases (PCVADs). In this way, the forms of presentation among the disease are classified into four types: (i) subclinical infection by PCV2 (PCV2-SI) where the pigs are clinically asymptomatic, which is the most common; (ii) PCV2 systemic disease (PCV2-SD) to replace PMWS, which is manifested by wasting, weight loss, enlarged lymph nodes, jaundice, and pallor; (iii) PCV2 reproductive disease (PCV2-RD), which induces the presentation of mummies, abortions, and stillbirths; and (iv) porcine dermatitis and nephropathy syndrome (PDNS) [18]. Vaccination has been the primary approach to control PCVADs, and its contribution to viral control is observed through a decrease in viral loads and its elimination [19,20]. Furthermore, vaccination has contributed to minimizing the lymphatic lesions associated with PCV2-SD and minimizing the effects induced by the virus and its coinfections [20]. In relation to productive parameters, vaccination against PCV2 has improved daily weight gain, decreased mortality, and reduced costs invested in medications [21]. However, although vaccination has enabled the management of PCVADs, it is observed that this approach needs to exert greater control over PCV2 for the following reasons: (i) the available vaccines do not confer sterile immunity [22], an observation that may contribute to the evolution of new genotypes [23] and variations in clinical manifestations—at present, the predominant presentation of PCVADs is PCV2-SI; (ii) traditional single-genotype PCV2 vaccines, while offering cross-protection, may not offer enough coverage to include evolving field viruses [24]; and (iii) the high genetic variability within PCV2 has resulted in PCV2 vaccines being less effective [25]. Additionally, it appears that minor variations accumulating at the ORF2 sequence level means that the number of T cell epitopes shared between PCV2a (particularly used as an immunogen) and field strains is decreasing [26], leading to impaired immune differentiation against PCV2, including the binding of neutralizing antibodies (Abs) [27].

Commercial PCV2 vaccines can be categorized into whole-virus-inactivated and subunit vaccines that use the viral Cap protein as the immunogen; both vaccine types induce neutralizing Abs and cell-mediated immunity (CMI) [20]. Beyond the type of vaccine against PCV2, there has been a broad discussion about its effectiveness. Therefore, there is a growing need to improve existing vaccines. When selecting vaccine candidates, it is essential to consider the T cell epitope content and density, as well as the potential to induce memory T cells that will recognize epitopes contained within circulating strains [26]. To study the above, computational tools have been developed and utilized to predict, among other aspects, the role of T cell epitopes in CMI, known as computational vaccinology [28]; this term incorporates epitope mapping, antigen selection, and vaccine construct design [29,30]. It is recognized that T cell epitopes are similar in vaccine and challenge strains, being a protective mechanism against PCV2 in the absence of cross-reactive Abs [31], and studying these epitopes may therefore allow us to predict the effectiveness of vaccines targeting PCV2. Algorithms have, thus, been developed to compare the T cell epitope content in sequences of vaccines and viral field strains, including Epitope Content Comparison (EpiCC) [32,33].

This study aimed to determine the prevalence of PCV2 in Colombia from 2020 to 2021, collecting swine samples from vaccinated herds exhibiting clinical signs compatible with PCVADs. Subsequently, the PCV2 genotypes detected were molecularly characterized. At the same time, the EpiCC tool established similarities and differences between the T cell epitopes for three vaccines and the Cap sequences from the circulating strains.

## 2. Materials and Methods

### 2.1. Sample Collection and Processing

This study scrutinized herds experiencing a history (routine) of vaccination against PCV2 (all vaccines available in Colombia correspond to the PCV2a genotype). These herds are located in Colombia’s five major swine-production provinces (Cundinamarca, Antioquia, Atlántico, Valle del Cauca, and Eje Cafetero). According to the density of sows per province (source: PorkColombia; https://porkcolombia.co/wp-content/uploads/2024/05/ED-DIGITAL-273-SEPT-OCT.pdf; accessed on 1 November 2023), the 84 herds under investigation were scrutinized proportionally between 2020 and 2021. The inclusion criterion for collecting samples was the clinical manifestation of PCVAD (PCV2-SI was excluded) according to the categorization previously suggested [18]. Under this criterion, we included 167 pigs suspected of clinical PCVADs. The distribution of pigs by geographic provinces, herds, and the sample type collected are described in Table 1.

According to the PCVAD categorization mentioned above, the pigs scrutinized were distributed as PCV2-RD (*n* = 38), PCV2-SD associated with PMWS (*n* = 10), PCV2-SD associated with respiratory disease (*n* = 138), and PCV2-PDNS (*n* = 4). Samples were collected from each pig, including lung (*n* = 160), spleen (*n* = 150), lymph node (*n* = 150), and serum (*n* = 147) (Table 1). The ages among the pigs under investigation were nursery pigs (aged 3 to 7 weeks, *n* = 131) and growing/finishing pigs (aged 9 to 22 weeks, *n* = 36). Tissue samples were suspended in sterile phosphate-buffered saline, homogenized, and centrifuged at 8000× *g* (Sorvall ST 8^®^ Thermo Scientific, Langenselbold, Germany) for 5 min. The serum samples were obtained via blood centrifugation at 3000× *g* for 10 min. The tissue’s supernatant and serum samples were subsequently stored at −80 °C until further processing at the Animal Virology Laboratory of the Facultad de Medicina Veterinaria y de Zootecnia, Universidad Nacional de Colombia, Bogotá. A portion of the collected tissues (lung, spleen, lymph node, and tonsil) was stored in 10% neutral buffered formalin and processed for histopathology via the hematoxylin and eosin protocol at the Pathology Laboratory, Facultad de Medicina Veterinaria y de Zootecnia, Universidad Nacional de Colombia, Bogotá.

### 2.2. Detection of PCV2 and Sequencing

According to the manufacturer’s instructions, DNA from serum samples and tissues was extracted using the high pure viral nucleic acid kit (Roche^®^, Ref 11858874001, Mannheim, Germany). All extractions were subsequently stored at −80 °C until further processing. As previously reported, individual detection of PCV2-DNA was conducted via end-point PCR employing specific primers [34]. Reactions were carried out in a total volume of 25 µL comprising 0.25 µL of Taq polymerase (5 U/µL) (Go taq flexi-Promega^®^, Ref M8295, Madison, WI, USA), 5X Taq buffer (2.5 µL), 2 mM MgCl2, 0.5 mM dNTPs, 1 µL of each primer (20 µM), and 2 µL of extracted DNA. The thermal cycling conditions for the PCR involved an initial denaturation step at 94 °C for 5 min, followed by 35 cycles of denaturation at 94 °C for 30 s, annealing at 58 °C for 30 s, extension at 72 °C for 45 s, and a final extension at 72 °C for 5 min. Reactions were carried out on a Biorad^®^-DNA (Hercules, CA, USA) thermocycler. The PCV2-positive status was observed by detecting a 505 bp band on 1% agarose gel. Subsequently, the positive samples were selected for the PCV2-ORF2 amplification using specific primers, as previously reported [35], which generated an amplicon of 685 bp. PCR assays for sequencing of PCV2-ORF2 were conducted in a total volume of 50 μL comprising 25 μL of Q5 High-Fidelity 2 × Master Mix (NEB, Ipswich, MA, USA), 1.25 μL (10 μM) of each primer, 2 μL DNA template of positive samples, and 20.5 μL ddH2O. PCR conditions comprised initial denaturation at 95 °C for 30 s, followed by 35 cycles of denaturation at 98 °C for 10 s, annealing at 57 °C for 30 s, extension at 72 °C for 40 s, and a final extension at 72 °C for 5 min. PCR products were visualized with 1% agarose gel. After amplification, PCR products were purified using the QIAquick PCR purification Kit^®^ (Qiagen, Hilden, Germany), according to the manufacturer’s instructions. Subsequently, the PCV2-ORF2 nt coding region sequencing was performed bi-directionally using the Sanger method at the commercial sequencing facility SSiGMol (Servicio de Secuenciación y Análisis Molecular), Instituto de Genética, Universidad Nacional de Colombia, Bogotá.

### 2.3. Sequencing and Phylogenetic Analysis of PCV2-Capsid Protein (ORF2)

In this study, we obtained 57 PCV2-ORF2 nt sequences and those compared with 45 other reference strains whose selection criterion was the coverage of genotypes PCV2a to PCV2h. We divided the PCV2 genotypes in accordance with the following criteria: when the ORF2 genetic distance between them was 0.035 and agreed with the distance detected between viral sequence groups in the phylogenetic trees [11]. All alignments were conducted with sequences available in the NCBI GenBank nt database and meticulously selected via Nucleotide BLAST (Basic Local Alignment Search Tool) and achieved by ClustalW using MEGA7 [36]. Phylogenetic analysis was conducted using the maximum-likelihood (ML) method and the Hasegawa-Kishino-Yano and discrete Gamma distribution (HKY + G) model established upon selecting the best-fit model based on nucleotide substitution base and the Bayesian information criterion (BIC) as implemented in MEGA7 [36]. The robustness of the ML trees was statically evaluated via bootstrap analysis with 1000 bootstrap samples. Information detailing the reference sequences used in this study is provided in Table S1.

### 2.4. PCV2 Real-Time PCR in Serum and Tissue Samples

To discriminate between PCV2a and PCV2b/d, we employed a real-time PCR assay that detects a section of the PCV2-ORF2 using specific primers and a probe previously reported [37]. As the PCV2b probe also binds to the PCV2d genome, PCV2b-positive results were interpreted as proof of the presence of PCV2b or PCV2d, or both. The differentiation between PCV2b and PCV2d was conducted via subsequent sequencing. Briefly, in the real-time PCR, a 187 bp segment from PCV2-ORF2 was amplified from a PCV2-positive sample. Subsequently, this amplicon was cloned into the TOPO-TA cloning (ThermoFisher Scientific^®^), according to the manufacturer’s instructions. The PCV2 recombinant plasmid was sequenced at the commercial sequencing facility SSiGMol (Servicio de Secuenciación y Análisis Molecular), Instituto de Genética, Universidad Nacional de Colombia, Bogotá. The reactions were carried out in a total volume of 20 μL, comprising 50 ng DNA, 10 μL of 1X LightCycler^®^ 480 probes master, 0.4 μM of each forward and reverse primer, and 0.25 μM of each probe. The reaction was conducted in a LightCycler 480^®^ Real-Time PCR (Roche, Burgess Hill, UK) instrument under thermal conditions involving an initial denaturation step at 95 °C for 10 min, followed by 40 cycles of denaturation at 95 °C for 15 s, annealing at 60 °C for 45 s, and a final extension step at 72 °C for 2 s (acquiring). Samples with Ct values ≤ 37 were considered positive. The negative control was ddH2O, and the positive control was the PCV2 plasmid. All reactions were conducted in triplicate. The viral genome copies were quantified using titrated plasmids comprising PCV2-ORF2. The 10-fold serial dilutions evaluated the limit of detecting real-time qPCR from 1 × 10^2^ to 1 × 10^9^ copies/μL. The quantification cycle (Cq) values ranged from 8.6 to 37 cycles with a linear correlation (R2) of 0.989 (slope = −3908) between the Cq value and the logarithm of the PCV2 copy numbers. Samples with no Ct at 37 cycles were considered negative. Viral concentrations were expressed as the mean log10 PCV2 genome copies/mL (lgc/mL) or log PCV2 genome copies/g (lgc/g). The specificity of the qPCR-PCV2 was corroborated by using samples positive for other DNA viruses, including PCV3, PCV1, and porcine parvovirus 1 and 2 (PPV1 and PPV2).

### 2.5. PCVAD Categorization

To classify samples according to viral load, we implemented the categorization previously suggested [38], and positive samples were stratified accordingly into two categories: (i) low-PCV2 (Ct > 25 to 37) and (ii) high-PCV2 (Ct < 25). This categorization was adopted to compare PCVAD rather than non-PCVAD pigs, considering high-PCV2 as PCVAD and low-PCV2 as non-PCVAD. According to the above, when a case was classified as PCVAD, it was corroborated via histopathology.

### 2.6. Histopathology

Sixty pigs from the total sampled (*n* = 167) were scrutinized via histopathology; the tissues analyzed were lung (*n* = 60), lymph nodes (*n* = 60), spleen (*n* = 56), and tonsil (*n* = 44). Tissues were fixed in 10% buffered formalin, subsequently embedded in paraffin, and routinely processed for histopathology. To categorize lesions in lymphoid tissues (tonsils, spleen, and lymph nodes), we used the scoring system previously proposed [39], while for lung lesions, microscopic pneumonia was categorized as bronchopneumonia, bronchointerstitial, and interstitial pneumonia. The lesions were classified into three levels, low, moderate, and severe, regardless of their characterization [39].

### 2.7. T Cell Epitope Content Comparison (EpiCC) Analysis

This analysis included 57 PCV2-Cap protein sequences obtained in this study (55 PCV2d and 2 PCV2a). Each sequence was compared to three vaccines using EpiCC to quantify T cell epitope relatedness, as previously described [33]. Initially, potential T cell epitopes were identified using PigMatrix [40], which parses sequences into 9-mers and assesses the binding potential of each 9-mer to 11 swine leukocyte antigen (SLA) class I alleles (SLA-1*04:01, 1 *08:01, 1*12:01, 1*13:01, 2*04:01, 2*05:01, 2*12:01, 3*04:01, 3*05:01, 3*06:01, and 3*07:01) and 5 SLA class II alleles (SLA-DRB1*02:01, 04:02, 06:02, 07:01, and 10:01). Subsequently, EpiCC was used to assess the relatedness of T cell epitopes contained in a PCV2-Cap protein of each Colombian strain and those in protein sequences of three vaccines: a bivalent PCV2a/PCV2b chimeric modified live virus vaccine (VacAB) and two PCV2a Cap sequences from subunit vaccines produced using baculovirus expression systems (VacAlt-a I and VacAlt-a II). The two PCV2a vaccines corresponded to PCV2 vaccines employed in the herds under investigation (in Colombia, there are only PCV2a vaccines available). In addition, all PCV2 vaccines marketed in Colombia are currently imported; that is, there are none manufactured in the country nor based on local strains. Higher EpiCC scores represented a greater relatedness; each PCV2-Cap protein sequence was also compared to itself to calculate an EpiCC baseline score, which assesses each sequence’s T cell epitope content density. T cell epitope content coverage percentage for each strain was calculated by dividing the EpiCC score by the corresponding strain baseline score.

### 2.8. Statistical Analysis

A statistical analysis compared viral loads between vaccine types and herd vaccination protocols. Data normality was assessed using the Shapiro–Wilk test. A conventional ANOVA test was then conducted with an alpha of *p* < 0.05, using the “PCR” package from the RStudio program version 4.3.0 [41]. A Tukey test was used to compare the means between each treatment, using the “Agricola” package of the RStudio program version 1.3-7.

## 3. Results

### 3.1. PCV2 Detection

**Figure 1:**
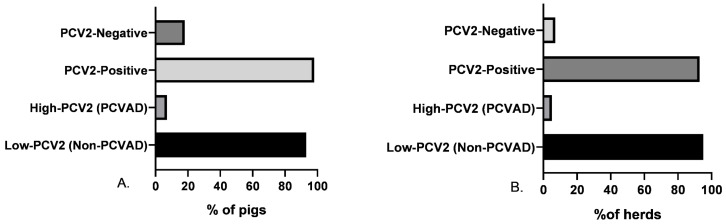
Prevalence via real-time PCR of PCV2 in pigs and herds. (**A**) PCV2 prevalence among pigs (total *n* = 167; PCV2-negative *n* = 30; PCV2-positive *n* = 137) and within PCV2-positive pigs, the percentages of high-PCV2 (PCVAD) and low-PCV2 (non-PCVAD) viral load are indicated. (**B**) PCV2 among herds (total *n* = 84; PCV2-negative *n* = 6; PCV2-positive *n* = 78) and within PCV2-positive herds, the percentages of high-PCV2 (PCVAD) and low-PCV2 (non-PCVAD) viral load are indicated. Pigs and herds were both categorized as PCV2-negative if PCV2-DNA was not identified; low-PCV2 if PCV2-DNA was under 5.19 log10 copies/mL or g for serum and the tissues, respectively, and high-PCV2 if it was above 5.20 log10 copies/mL or g for serum and the tissues, respectively.

**Figure 2:**
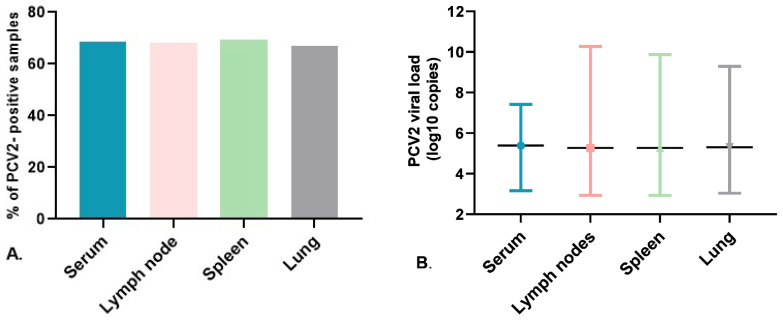
Detection of PCV2 in serum samples (*n* = 147), lymph nodes (*n* = 150), spleen (*n* = 150), and lungs (*n* = 160). (**A**) Prevalence via real-time PCR in samples. (**B**) PCV2 viral loads (log10 copies/mL or g for serum and the tissues, respectively) in the collected samples; the horizontal bars correspond to the average viral load within the sample type, and the vertical bars correspond to the minimum and maximum values of viral loads identified.

In this study, the condition for recruiting each of the 84 participating herds was that they exhibited a routine vaccination program against PCV2, regardless of the type of vaccine used. Thus, by type of vaccine in herds, the subunit vaccine was the most commonly employed, at 73.8% (62/84), followed by the chimeric vaccine, at 20.2% (17/84), while the whole virus inactivated was the third most commonly used, at 6% (5/84). Among the 167 clinically suspected PCVAD pigs scrutinized, 124 were immunized with the subunit, 34 with the chimeric, and 9 with the whole virus inactivated vaccine. In turn, 82% of the pigs (137/167) were PCV2-positive, while 18% (30/167) were negative. Among the PCV2-positive pigs, only 7% (10/137) exhibited a high-PCV2 load (5.20 to 9.28 lgc/mL, i.e., PCVAD), while 93% (127/137) exhibited a low-PCV2 load (3.2 to 5.19 lgc/mL, i.e., non-PCVAD; see Figure 1A). Discriminating by herd, 92.9% (78/84) were positive and 7.1% were PCV2-negative (Figure 1B); by sample, 68.7% (101/147) of the serum samples were PCV2-positive, with 66.9% (107/160) in lung tissue, 68% (102/150) in lymph nodes, and 69.3% (104/150) in the spleen (Figure 2A).

PCV2-DNA was identified simultaneously in the same pig in 45% (75/167) of the four samples scrutinized (lung, spleen, lymph nodes, and serum), 29% (48/167) for three samples, 13% (22/167) for two samples, and 13% (22/167) in a single sample. Regarding the viral loads, the percentage of PCV2-negative samples (Ct values ≥ 37) was similar among the four types of samples, ranging between 30 and 33%; the average viral loads among the four samples included low-PCV2 loads (below 5.20 lgc/mL or g) (Figure 2B). Analyzing by province (*n* = 5), all were PCV2-positive. In the Cundinamarca and Atlántico provinces, all the herds scrutinized were PCV2-positive, while in the remaining (Antioquia, Valle del Cauca, and Eje Cafetero), the proportion of PCV2-positive herds was above 80%. The highest viral loads (higher than 9.0 lgc/mL) were identified in Cundinamarca and Antioquia provinces (Table 2).

**Figure 3:**
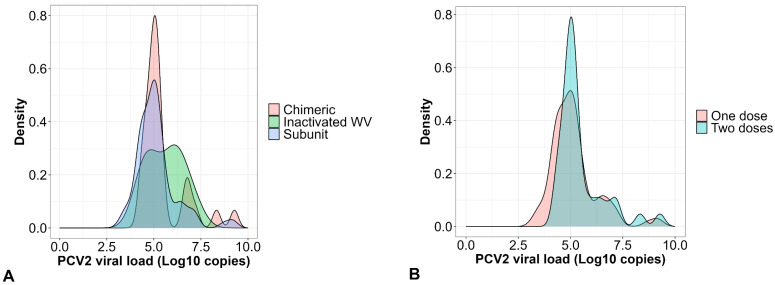
PCV2 viral loads (log10 copies/mL or g for serum and the tissues, respectively) in pigs from herds vaccinated against PCV2 in Colombia. (**A**) PCV2 viral loads associated with the type of vaccine applied against PCV2 [chimeric (*n* = 34); inactivated whole virus (WV) (*n* = 9) and subunit (*n* = 124)]. (**B**) PCV2 viral loads associated with the two vaccination protocols [one dose (*n* = 124) and two doses (*n* = 43)] against PCV2 employed in Colombia.

When analyzed by the type of vaccine employed (subunit, inactivated whole virus, and chimeric) in Colombian herds, the average PCV2 viral load remained below 5.20 lgc/mL, corresponding to low-PCV2 loads. We revealed no statistical difference between vaccines (*p* = 0.177). However, it is pertinent to note that high-PCV2 loads (≥7.5 lgc/mL or g) were detected in particular pigs, reaching loads of up to 10^9^, particularly with the chimeric and the subunit vaccine (Figure 3A). Regarding the vaccination protocol used on the herd, there were no statistical differences (*p* = 0.132) between applying one or two doses. However, we identified higher viral loads (greater than 9.0 lgc/mL) in pigs that received a single dose at weaning (Figure 3B).

**Figure 4:**
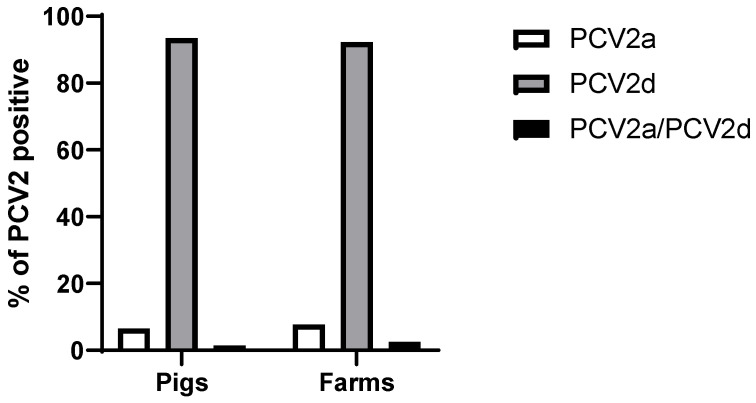
Prevalence of the PCV2 genotypes in the pigs (*n* = 137) as well as in the herds (*n* = 78) that were PCV2-positive. The presence of PCV2 is indicated in the form of single infections (a single genotype) and coinfections (at least two genotypes in the same pig).

To establish the PCV2 genotypes in the 137 PCV2-positive pigs, we used previously reported primers [42], which differentiate between PCV2a and PCV2b/d; in other words, PCV2-positive pigs that were not PCV2a, so they were PCV2b or PCV2d. Subsequently, ORF2 was sequenced to differentiate between the latter two genotypes. The findings established that PCV2d was the predominant genotype in pigs with 93.4% (128/137), while in the herds, it was 92.3% (72/78). The other genotype identified was PCV2a, present in 6.6% (9/137) of pigs and 7.7% (6/78) of herds; this genotype was only detected in the Valle del Cauca province. Additionally, within these PCV2a-positive herds, we identified two pigs with PCV2a/PCV2d coinfection, corresponding to 1.5% (2/137) (Figure 4).

### 3.2. Sequence Analysis of PCV2

**Figure 5:**
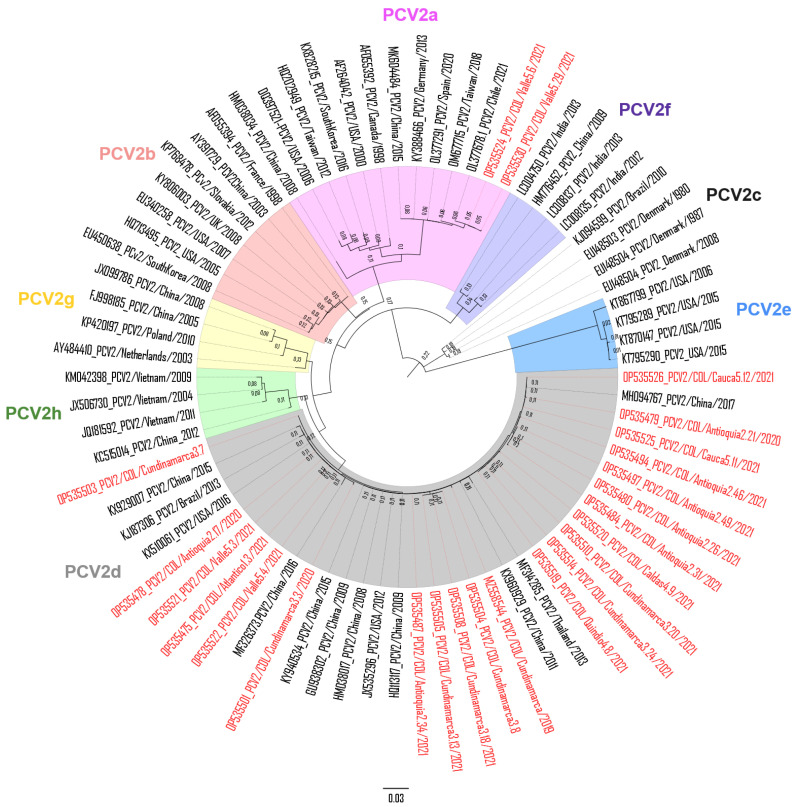
Maximum likelihood phylogenetic tree of 22 PCV2-ORF2 sequences determined in Colombia (2 PCV2a and 20 PCV2d), inferred based on the alignment among the nucleotide sequences. The tree was constructed via ML analysis using the Tamura 3 parameter with gamma distribution, and tree topology was evaluated with 1000 bootstrap replicates. The sequences in red font corresponded to the Colombian ones determined in this study and were juxtaposed with 53 sequences available in the NCBI GenBank nucleotide database.

From the 137 PCV2-positive pigs, 57 PCV2-ORF2 sequences were acquired. Their traits in terms of province of origin, collection date, and GenBank access number are indicated in Table S2. Multiple alignments between the 57 Colombian sequences revealed an identity for nt ranging from 88.2 to 100%, while for amino acids (aa), it was 84.2 to 100%. When the Colombian scrutinized sequences were juxtaposed with PCV2-ORF2 sequences (*n* = 53) available in the GenBank, the nt identity ranged from 83 to 100%, while for aa, it was 76.4 to 100%. Among the 57 Colombian sequences retrieved, 96.5% (55/57) were classified as PCV2d, while 3.5% (2/57) were PCV2a (Figure S1). The phylogenetic tree in Figure 5 shows the distribution of 20 PCV2d and 2 PCV2a Colombian selected sequences from this study juxtaposed with reference sequences of the PCV2a to PCV2h genotypes. The comparison of Colombian strains with those from PCV2 genotypes available in the NCBI GenBank database is indicated in Table 3.

The PCV2 aa sequence comparisons between the 57 strains from Colombia and the reference ones revealed greater diversity among the PCV2a genotype, with an identity ranging from 90.8 to 100%. Notably, Colombian PCV2a sequences differed by up to 10% from reference sequences reported in 2000 (AF264042-USA) and exhibited a higher identity with recently detected sequences from Chile 2021 (OL377678), Spain 2020 (OL377291), and Germany 2015 (KY388466). We determined 25 substitutions at the PCV2a-Cap level in our sequences juxtaposed with 11 ORF2-Cap reference aa sequences identified worldwide. Concerning Colombian PCV2d strains (*n* = 55) juxtaposed with 14 reference sequences, three aa substitutions (F8Y, A133S, G/R169G/R) were determined (Table S3). Notably, changes in Colombian PCV2a-Cap aa sequences agreed with the most frequent ones reported in vaccinated and unvaccinated PCV2 pigs [42], making it impossible to determine whether they correspond to vaccine-derived variants.

### 3.3. Histopathology

Four tissues (lung, lymph nodes, spleen, and tonsil) from 60 PCV2-positive pigs were processed for histopathological analysis. Regarding the lungs, we analyzed the patterns of pneumonia, revealing that there were three types: suppurative bronchopneumonia in 45% (27/60) of the cases, interstitial pneumonia in 35% (21/60), and bronchointerstitial pneumonia in 10% (6/60). The histopathological findings were classified into three scores (low, moderate, and severe) according to severity (Table S4); in the lung, the most common lesion with a severe score was exudation in airways at 33% (20/60), while the moderate and low scores corresponded to the thickening of alveolar septa, with 56.6% (34/60) and 25% (15/60), respectively. Concerning lymph nodes, neutrophils in the sinus system received the highest severe score with 16.6% (10/60), while moderate and low scores were assigned to follicle lymphoid depletion, with 28.3% (17/60) and 31.6% (19/60), respectively. Concerning the spleen, the most common lesion in the three scores was mixed lymphoid depletion, with 12.5% (7/5), 30.3% (17/56), and 30.3% (17/56), respectively. Finally, in the tonsils, lymphoid depletion was the most common lesion presented in the three scores.

### 3.4. EpiCC Analysis of T Cell Epitope Relatedness and Coverage

**Figure 6:**
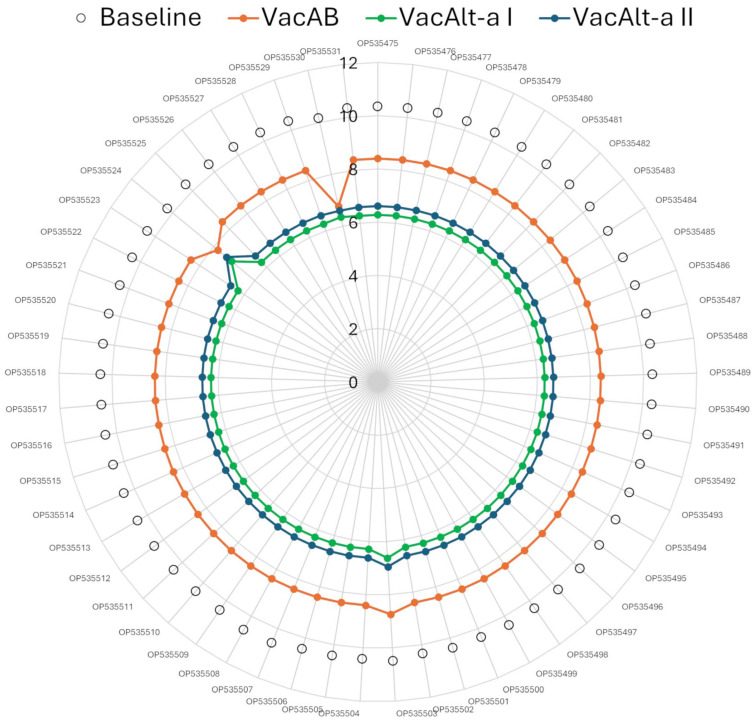
EpiCC scores for three vaccines targeting 57 Colombian strains. Each axis corresponds to the PCV2 Cap of one virus. Axis labels are NCBI GenBank database accession numbers. Baseline EpiCC scores for each strain are represented as open circles. EpiCC scores for each vaccine are indicated in distinct colors. Strains are sorted by province and collection date.

The mean EpiCC scores and T cell epitope coverage are indicated in Table 4. Concerning each vaccine/strain comparison, EpiCC scores were compared and visualized using a radar plot (Figure 6). For all the comparisons, VacAB exhibited higher EpiCC scores and T cell epitope coverage than monovalent vaccines. Overall, the mean baseline EpiCC score was 10.38, with a minimal variation, suggesting that T cell epitope content densities are similar among Cap protein sequences of Colombian strains. The overall mean EpiCC score for VacAB against the strains was 8.36, compared to 6.30 and 6.63 for VacAlt-a I and VacAlt-a II, which translates to a mean of 80.6% T cell epitope coverage for VacAB, compared to 60.8% and 63.9% for VacAlt-a I and VacAlt-a II. Similarly to baseline scores, except for the two PCV2a strains, EpiCC scores for PCV2d strains were also constant, suggesting that the predicted T cell epitope content in the strains was highly similar. As expected, the EpiCC scores and T cell epitope coverage were also similar among provinces. VacAB’s highest mean T cell epitope coverage was for Antioquia (81.1%). The findings displayed that VacAB exhibited a broader potential T cell epitope coverage for this set of Colombian strains compared to the monovalent vaccines. Tables S5 and S6 show the values obtained from the individual (each sequence) and by geographic provinces of EpiCC and T cell epitope analyses.

## 4. Discussion

In this study, we determined the presence of PCV2 in 84 pig herds located in Colombia’s five major swine-production provinces, with a prevalence of PCV2 greater than 80% in both pigs and herds. This finding varies from previously established Colombian prevalences of 9.1% and 56.2% from the feces of clinically asymptomatic pigs, in 2015 and 2019, respectively [17], and 53% identified in gilts aged 180 to 200 days [43]. Although the prevalence of PCV2 in this study was high, the association between viral loads (low-PCV2 and high-PCV2) and PCVAD indicated that only 7% of the pigs presented high-PCV2 (PCVAD). This suggests that the clinical manifestations suspicious for PCVAD assumed as a condition for inclusion in the study could correspond to the effect of various factors, including the presence of other pathogens.

The vaccines against PCV2 currently marketed in Colombia are monovalent, using PCV2a-Cap in subunits or as inactivated whole virus. The findings corroborate that regardless of the type of vaccine, they provide control of viral loads as these were, on average, low in both pigs and herds (<10^3^, 5.5 lgc/mL or Ct ≥ 25). Second, these findings were similar to results obtained in the field (herd conditions) and controlled (experimental) conditions. In both cases, it has been demonstrated that vaccination against PCV2 reduces the viral load to levels where productive parameters are unaffected but does not prevent the presentation of PCVAD, particularly PCVAD-SI [35,44,45,46,47]. However, contradicting the viral load control concept, our study specifically detected vaccinated pigs with high loads (≥10^6^, Ct ≤ 20, 9.8 lgc/mL) regardless of the type of vaccine used. The reason for these findings cannot be inferred from this investigation, but distinct hypotheses can be suggested. The first would be a failure in the vaccination protocol, where some pigs did not receive the vaccine and were more susceptible to infection. The second is that all the pigs received the vaccine. Regarding this last proposition, there are prior studies in serum samples of vaccinated pigs where high viral loads (>6.0 log_10_ copies/mL) were identified [46]. For instance, one study conducted on vaccinated breeding and fattening herds in China (2022) detected >15% of positive samples in growing/finishing pigs and gilts exhibiting viral loads >10^6^, while 8.3% of growing/finishing pigs exhibited viral loads >10^8^ [48]. In another study conducted under experimental conditions in pigs vaccinated against PCV2 at different weeks of age (3, 6, and 10), the highest viral loads (4.9 lgc/mL) were observed in pigs vaccinated at week 10, indicating the presence of an immunological window induces by the decrease in maternal immunity and also that the best vaccination protocol was at 3 and 6 weeks of age [35]. The latter was corroborated in this study, where the herds under investigation were vaccinated at three weeks of age. However, the highest viral loads were identified in herds employing the single-dose protocol compared to those that used two doses, underscoring that this latter protocol may confer extensive protection in terms of viral loads and probably reduce the occurrence of PCVADs.

Another contribution of this study is the heterogeneous viral loads obtained in samples from the same individual. In this sense, our study identified the lowest viral loads in serum samples and lungs. While they were higher in lymphoid tissue, 8% of the spleens and 9.3% of the lymph nodes exhibited high viral loads, confirming both tropism and preferential replication of PCV2 in lymphoid tissue. The latter has been demonstrated by several prior investigations on PCV2 pathogenesis, where its productive replication in T lymphoblasts was demonstrated [49,50], and others on PCV2 evolution displayed that it exhibited improved adsorption to T cells [51]. From a diagnostic perspective, PCV2 is routinely detected from serum samples where it is expected to find low viral loads; we recommend considering that the viral load heterogeneity in samples may obstruct its control in herds. Additionally, our study corroborates other findings [25], where the PCV2 prevalence among serum samples was low (11%). These sera with low viral loads are usually interpreted as an adequate control conferred by the vaccine; however, the virus may be in an active replication state with high viral loads in the lymphoid tissue and may eventually manifest as a PCVAD.

Regarding PCV2 genotypes, PCV2d is currently predominant in Colombia and is widely distributed in the five provinces studied. However, PCV2a is present in a low proportion (6.6%) in one province (Valle del Cauca), detected in mono-infection and PCV2a/PCV2d coinfection. A concurrence between PCV2 and other swine viruses has been established and widely reported worldwide, while coinfections between PCV2 genotypes are rare; three investigations have reported this in vaccinated herds, one in Poland, where they identified PCV2a/PCV2b and PCV2a/PCV2d coinfections [46], and two others in China, where they identified PCV2d/PCV2b and PCV2d/PCV2e [52] and another PCV2b/PCV2d [52] coinfection. The implications of these coinfections are not defined; however, it has been suggested that they may contribute to extended severe signs of PCVADs [53] and that coinfections with genotypes may facilitate the recombination between PCV2 genomes [54], and there is consensus that PCV2 exhibits a high rate of substitution and recombination events [25]. In this sense, the findings of this study reveal that PCV2 in Colombia exhibits similar evolutionary patterns compared with other countries, in that until 2014, the predominant genotypes were PCV2b (82%) and PCV2a (18%) [16], where PCV2d now predominates [17]. However, as mentioned above, PCV2a has remained in one province of the country but with abundant patterns of change. Comparing our PCV2a-ORF2 nt sequences with others, we identified the highest identity with PCV2a strains previously detected in Chile (2021), coinciding with a substantial number of substitutions at the capsid protein that could eventually affect both the virus’s tropism and the immune response against it. Regarding tropism, it has been reported that two regions outside the Cap protein are involved in binding to the glycosaminoglycan receptor; the first corresponds to aa 59, 206, and 63 (A/K/S), and the second at aa 57, 68, and 134 (V/A/T) [55]. However, regarding the immune response, two distinct substitutions in aa 68 and 133 (A/A) were identified in Cap, which has been suggested for a poorly diversified viral selection [56] and may facilitate escape from vaccine immunity [57]. Likewise, it was demonstrated that mutations at position aa 133 of Cap impact cross-reactivity with monoclonal Abs [22]. In the PCV2a strains detected in this study, we identified in the Cap sequence the canonical pattern for aa 59, 206, and 63 [A/K(S)]. However, we identified two distinct mutations in aa 57, 68, and 134 [V/S(P)], and two substitutions at aa 68 and 133 (S/V). These findings suggest that in Colombian PCV2a strains, adsorption and viral antigenicity may be altered, the implications of which warrant further investigation. Additionally, it was impossible to determine whether these strains correspond to vaccine-derived variants since aa sequence changes in Cap showed profiles of PCV2a variants found in both vaccinated and unvaccinated pigs [42].

There is consensus that histopathological lesions associated with PCV2 decrease in PCV2-vaccinated pigs [58], and that they are even lower if the vaccine comprises the same circulating genotype [59]. Additionally, the associated microscopic lesions are imperceptible, moderate to mild, when the pig has a PCV2 mono-infection, while they worsen in coinfections [60]. In our study, the most common clinical presentation was PCVAD-SD in the respiratory form. The histopathology among the lungs displayed pneumonia patterns in the following order: suppurative bronchopneumonia, interstitial pneumonia, and bronchointerstitial pneumonia. These patterns were similar to those reported previously [58], although in this study, interstitial pneumonia emerged as the most predominant, while the most severely scoring injury was airway exudation. The lesions found were non-specific for PCV2, including moderate-to-severe peri-bronchiolar and perivascular infiltration, linked to coinfections with mycoplasma [61]. However, no lesions associated with coinfection with PRRSV were detected, including severe hemorrhages, emphysema, sarcoid changes, or alveolar spaces comprising necrotic debris [62]. In the lymph nodes, the most severe lesion was neutrophils in the sinus system, but follicle lymphoid depletion emerged as the most common in low and moderate scores. This form has been identified in severe scores in PCV2/PRRSV coinfections [62] and moderate to severe in PCV2/*Mycoplasma hyopneumoniae* coinfections [60]. Therefore, the histopathological findings found in this study cannot be explicitly attributed to PCV2 infection.

The development of immunoinformatic tools, including EpiCC, is generating rapid advances in computational vaccinology in humans [63,64] and veterinary medicine [26,33,40,65]. Ideally, a vaccine should induce memory T cells that recognize the epitopes contained in the circulating strains; that is, the epitope content of a vaccine should be similar among the circulating recognition strains to generate broad immunity and protection [32]. In this study, we employed EpiCC to predict T cell epitopes for the 57 Cap sequences acquired from Colombia and compared them with three vaccines. The vaccines under investigation were predicted to cover extended T cell epitope content in PCV2d strains than in PCV2a strains. Consistently with the high similarity between PCV2d sequences, the content of shared T cell epitopes was also very similar in the five provinces studied. The greater variability identified for the two PCV2a strains in this study coincides with other reports [33] determining high variability for PCV2a and PCV2b with patterns of change by geographical areas. In this sense, EpiCC analyses on PCV2a strains in South America have only been conducted with strains from Chile [33], determining the lowest scores, similarly to that identified for Colombia in this study. On the other hand, the PCV2d strains acquired in this study displayed a minimal intragenotype variability similar to that reported in China [13], and the recent appearance and spread of PCV2d explain this.

The findings presented in this study corroborate the proposal of [26,33], wherein the use of a bivalent vaccine (VacAB: PCV2a and PCV2b-ORF2 sequences) increased the average coverage of T cell epitopes against the field strains selected. In our research, VacAB improved the average coverage in the five provinces studied by 19.8% and 16.7% compared to the two PCV2a vaccines. Likewise, VacAB increased the EpiCC score, equivalent to 2.1% and 1.7% additional epitopes covered compared to the two monovalent PCV2a vaccines, higher than the 1.3% reported previously [33]. It should be noted that the lowest coverage scores were in the Valle del Cauca province, where we identified the two PCV2a strains exhibiting the greatest variations in Cap.

The EpiCC results exhibited immunological distinction between routinely used PCV2 vaccines (PCV2a for Colombia) and the field strains identified. The absence of sterile immunity against PCV2 involves factors beyond epitope specificity and immune response, but they should be part of PCVAD control strategies. The association between EpiCC score and vaccine efficacy has already been studied in pigs for influenza A, where higher scores predicted efficacy [32]. In the case of PCV2, prior investigations have suggested that vaccination with a homologous genotype might be more successful than with a heterologous genotype [19,59]. Higher EpiCC scores not only suggest a higher probability of vaccine success, but also a lower risk of failure in the future if the virus continues to change. This is noteworthy for PCV2 due to its extended mutation rate and the fact that vaccines do not induce sterile immunity, instead favoring the conditions for viral evolution. Therefore, using multi-genotype vaccines may be an approach to slow the emergence of variants that escape specific immunity, which has been suggested for other viruses [66]. Specifically, the high prevalence of PCV2d strains in Colombia suggests that a bivalent or trivalent vaccine that includes this genotype should be developed.

## 5. Conclusions

In conclusion, this study confirmed that the PCV2d genotype is the most prevalent in Colombia and that both the vaccination protocols implemented and the commercialized vaccines (PCV2a) contribute to reducing the presence of PCVADs through maintaining low viral loads. However, they do not eliminate the virus, likely favoring the generation of variants. Additionally, the computational vaccinology tool EpiCC established that polyvalent PCV2 vaccines confer a better coverage of T cell epitopes for the PCV2 strains circulating in Colombia.

## Figures and Tables

**Table 1:** Description of the pigs under investigation to detect PCV2 with clinical signs of PCVADs according to their geographical distribution, herds, and collected samples.

Province	Sow Density Population	Number of Herds	Number of Pigs Scrutinized	Serum	Lung	Lymph Node	Spleen
Cundinamarca	34,646	11	25	22	25	23	23
Antioquia	143,821	38	69	57	63	58	58
Atlántico	18,910	5	10	8	9	8	8
Valle del Cauca	56,883	20	39	37	39	37	37
Eje Cafetero	30,175	10	24	23	24	24	24
Total	284,435	84	167	147	160	150	150

**Table 2:** Detection of PCV2 in the five provinces scrutinized by samples, farm, and viral load ranges.

Province	% Positive Samples (Positive/All Tested)	% Positive Herds (Positive/All Tested)	Viral Loads (log10 Copies/mL or g for Serum and the Tissues, Respectively) Minimum–Maximum, Mean
Cundinamarca	100 (25/25)	100 (11/11)	4.3–9.28, 5.68
Valle	82.1 (32/39)	90 (18/20)	3.2–8.68, 5.08
Antioquia	82.6 (57/69)	92.1 (35/38)	3.57–9.07, 5.2
Eje Cafetero	66.6 (16/24)	80 (8/10)	3.99–5.5, 4.75
Atlántico	70 (7/10)	100 (5/5)	4.41–5.17, 4.9
Total	82 (137/167)	92.8 (78/84)	3.2–9.28, 5.19

**Table 3:** Percentage of identity between the PCV2a and PCV2d-ORF2 sequences of nucleotides and amino acids among the Colombian strains identified in this study and PCV2a to PCV2h-ORF2 sequences available in the NCBI GenBank database.

		PCV2a (AF055392)	PCV2b (AF055394)	PCV2c (EU148503)	PCV2d (HM038017)	PCV2e (KT795289)	PCV2f (LC008135)	PCV2g (AY484410)	PCV2h (JX506730)
Identity ORF2 (nucleotide)	Colombian PCV2a sequences	93.7–100	90–90.2	85.8–86.2	88.1–89	80.9–81.1	90.2–90.3	90.3	91
	Colombian PCV2d sequences	90.2–91	92.9–93.7	88.8–89.4	99.3–100	83–84.3	91.9–92.8	96.2–96.9	94.2–94.9
Identity ORF2 (aa)	Colombian PCV2a sequences	90.8–100	85.4–86.4	79–80	84.7–84.8	76.4–76.6	85.1–86.2	86.1–87.8	86.7–86.9
	Colombian PCV2d sequences	89–91.1	92.6–95	85.2–86.8	99.1–100	79.3–80.5	91–94.4	94–95.9	95.5–96.4

**Table 4:** Mean EpiCC scores and T cell epitope coverage for each vaccine.

Province	Number of Sequences	EpiCC Scores				T Cell Epitope Coverage %		
		Baseline	VacAB	VacAlt-a I	VacAlt-a II	VacAB	VacAlt-a I	VacAlt-a II
All	57	10.38 (10.16–10.59)	8.36 (6.77–8.74)	6.30 (6.28–7.13)	6.63 (6.61–7.38)	80.6 (66.62–83.24)	60.8 (59.32–69.65)	63.9 (62.42–72.05)
Atlántico	1	10.35	8.40	6.28	6.61	81.1	60.7	63.9
Antioquia	23	10.35 (10.35–10.43)	8.40 (8.40)	6.28 (6.28)	6.61 (6.61)	81.1 (80.55–81.14)	60.7 (60.25–60.7)	63.9 (63.40–63.87)
Cundinamarca	17	10.44 (10.35–10.59)	8.42 (8.40–8.74)	6.30 (6.28–6.63)	6.63 (6.61–6.95)	80.7 (79.31–83.24)	60.4 (59.32–63.09)	63.5 (62.42–66.21)
Eje Cafetero	5	10.35 (10.16–10.43)	8.40 (8.40)	6.28 (6.28)	6.61 (6.61)	80.6 (80.55)	60.3 (60.25)	63.4 (63.40)
Valle del Cauca	11	10.32 (10.16–10.35)	8.19 (6.77–8.40)	6.37 (6.28–7.13)	6.68 (6.60–7.38)	79.4 (66.62–81.14)	61.7 (60.7–69.65)	64.7 (63.87–72.05)

Minimum and maximum values are indicated in parentheses.

## Data Availability

All required data are available as texts and figures in the main text of the article. The sequence datasets are publicly available at NCBI GenBank database.

## Supplementary Materials

The following supporting information can be downloaded at: https://www.mdpi.com/article/10.3390/vaccines12101119/s1, Table S1: Reference genomic sequences (*n* = 53) of porcine circovirus 2, (PCV2) available in GenBank, are used to juxtapose with the sequences retrieved in this study. Table S2: Traits of the PCV2-ORF2 sequences (*n* = 57) obtained from the 137 PCV2-positive cases from different provinces of Colombia. Table S3: Comparisons of amino acid sequences between PCV2a-Cap and PCV2d-Cap of the Colombian strains in this study and reference strains are available in the GenBank database. Table S4: Scores of histopathological lesions in collected tissues (lung, lymph node, spleen, and tonsil) from pigs with qPCR-PCV2 positive and with symptoms of PCVADs. Table S5: EpiCC analysis and T cell epitope coverage of the PCV2-ORF-2 of the 57 sequences retrieved from Colombia. Table S6: EpiCC scores and T cell epitope coverage obtained on three vaccines under investigation and on 57 PCV2-ORF2 sequences classified according to each geographic province scrutinized. Figure S1. Maximum likelihood phylogenetic tree of 57 PCV2-ORF2 sequences identified in Colombia, inferred based on the alignment of the nucleotide sequences. The tree was constructed via ML analysis using the Tamura 3 parameter with gamma distribution, and the tree topology was evaluated with 1000 bootstrap replicates. Colombian sequences generated in this study are highlighted in red and were juxtaposed with 53 sequences available in the NCBI GenBank nucleotide database.
