# Supplementary material for: Detection of PCV2d in Vaccinated Pigs in Colombia and Prediction of Vaccine T Cell Epitope Coverage against Circulating Strains Using EpiCC Analysis
Source: Vaccines (Basel). 2024 Sep 29;12(10):1119. doi: 10.3390/vaccines12101119 (PMC11511301; doi:10.3390/vaccines12101119)
Supplement: Supplementary file 1 [file vaccines-12-01119-s001.zip › vaccines-3079807-supplementary.pdf]

Table S1. Reference genomic sequences (n = 53) of porcine circovirus 2 (PCV2) available in GenBank and used to juxtapose with the sequences retrieved in the current study.

Number	genbank	Country	Collection year
1	KJ187306	Brazil	2013
2	JX535296	USA	2012
3	HM038017	China	2008
4	AY484410	Netherlands	2003
5	AF055394	France	1998
6	EU340258	USA	2007
7	AF055392	Canada	1998
8	AF264042	USA	2000
9	HM038034	China	2008
10	HQ202949	Taiwan	2012
11	KX828215	SouthKorea	2016
12	KP768478	Slovakia	2012
13	EU450638	SouthKorea	2008
14	KY806003	UK	2008
15	KJ094599	Brazil	2010
16	EU148503	Denmark	1980
17	KC515014	China	2012
18	MF314285	Thailand	2013
19	KX960929	China	2011
20	KT867799	USA	2006
21	KT870147	USA	2015
22	KT795289	USA	2015
23	LC004750	India	2013
24	LC008137	India	2013
25	LC008135	India	2012
26	KP420197	Poland	2010
27	FJ998185	China	2005
28	JX099786	China	2008
29	JQ181592	Vietnam	2011
30	JX506730	Vietnam	2004
31	KM042398	Vietnam	2009
32	EU148504	Denmark	1987
33	GU938302	China	2009
34	KY940534	China	2015
35	HQ113117	China	2009
36	AY391729	China	2003
37	DQ397521	USA	2006
38	EU148504	Denmark	2008

39	HM776452	China	2009
40	HQ713495	USA	2005
41	KT795290	USA	2015
42	KX510061	USA	2016
43	KX929007	China	2015
44	MF326373	China	2016
45	MH094767	China	2017
46	MZ558544	Colombia	2019
47	MZ558544	Colombia	2015
48	MZ558544	Colombia	2015
49	MK604484	China	2015
50	KY388466	Germany	2013
51	OL377291	Spain	2020
52	OM677715	Taiwan	2020
53	OL377678	Chile	2021

Table S2. Traits of the PCV2-ORF2 sequences (*n* = 57) obtained from the 137 PCV2-positive cases from different provinces of Colombia.

Genbank accession number	Region	Collection date	Symptoms	PCV2 genotype
OP535475	Atlántico	2021	Wasting /Dyspnea, cough	PCV2d
OP535476	Antioquia	2020	Dyspnea, cough	PCV2d
OP535477	Antioquia	2020	Dyspnea, cough	PCV2d
OP535478	Antioquia	2020	Dermatitis/ Dyspnea, cough	PCV2d
OP535479	Antioquia	2020	Dyspnea, cough	PCV2d
OP535480	Antioquia	2021	Wasting /Dyspnea, cough	PCV2d
OP535481	Antioquia	2021	Wasting /Dyspnea, cough	PCV2d
OP535482	Antioquia	2021	Stillbirth	PCV2d
OP535483	Antioquia	2021	Wasting /Dyspnea, cough	PCV2d
OP535484	Antioquia	2021	Wasting	PCV2d
OP535485	Antioquia	2021	Wasting	PCV2d
OP535486	Antioquia	2021	Dyspnea, cough	PCV2d
OP535487	Antioquia	2021	Dyspnea, cough	PCV2d
OP535488	Antioquia	2021	Wasting /Dyspnea, cough	PCV2d
OP535489	Antioquia	2021	Wasting /Dyspnea, cough	PCV2d
OP535490	Antioquia	2021	Wasting /Dyspnea, cough	PCV2d
OP535491	Antioquia	2021	Dyspnea, cough	PCV2d
OP535492	Antioquia	2021	Dyspnea, cough	PCV2d
OP535493	Antioquia	2021	Dyspnea, cough	PCV2d
OP535494	Antioquia	2021	Dyspnea, cough	PCV2d
OP535495	Antioquia	2021	Dyspnea, cough	PCV2d
OP535496	Antioquia	2021	Dyspnea, cough	PCV2d
OP535497	Antioquia	2021	Dyspnea, cough	PCV2d
OP535498	Antioquia	2021	Dyspnea, cough	PCV2d
OP535499	Cundinamarca	2020	Wasting /Dyspnea, cough	PCV2d
OP535500	Cundinamarca	2020	Wasting /Dyspnea, cough	PCV2d
OP535501	Cundinamarca	2020	Wasting /Dyspnea, cough	PCV2d
OP535502	Cundinamarca	2020	Wasting /Dyspnea, cough	PCV2d
OP535503	Cundinamarca	2020	Wasting /Dermatitis	PCV2d
OP535504	Cundinamarca	2020	Wasting /Dyspnea, cough	PCV2d
OP535505	Cundinamarca	2021	Dyspnea, cough	PCV2d
OP535506	Cundinamarca	2021	Dyspnea, cough	PCV2d
OP535507	Cundinamarca	2021	Dyspnea, cough	PCV2d
OP535508	Cundinamarca	2021	Dyspnea, cough	PCV2d
OP535509	Cundinamarca	2021	Dyspnea, cough	PCV2d
OP535510	Cundinamarca	2021	Dyspnea, cough	PCV2d
OP535511	Cundinamarca	2021	Wasting	PCV2d
OP535512	Cundinamarca	2021	Wasting	PCV2d
OP535513	Cundinamarca	2021	Dyspnea, cough	PCV2d
OP535514	Cundinamarca	2021	Dyspnea, cough	PCV2d
OP535515	Cundinamarca	2021	Dyspnea, cough	PCV2d
OP535516	Eje Cafetero	2021	Dyspnea, cough	PCV2d
OP535517	Eje Cafetero	2021	Dyspnea, cough	PCV2d
OP535518	Eje Cafetero	2021	Dyspnea, cough	PCV2d
OP535519	Eje Cafetero	2021	Dyspnea, cough	PCV2d
OP535520	Eje Cafetero	2021	Dyspnea, cough	PCV2d
OP535521	Valle del Cauca	2021	Wasting /Dyspnea, cough	PCV2d
OP535522	Valle del Cauca	2021	Wasting /Dyspnea, cough	PCV2d

OP535523	Valle del Cauca	2021	Wasting /Dyspnea, cough	PCV2d
OP535524	Valle del Cauca	2021	Wasting /Dyspnea, cough	PCV2a
OP535525	Valle del Cauca	2021	Dyspnea, cough	PCV2d
OP535526	Valle del Cauca	2021	Dyspnea, cough	PCV2d
OP535527	Valle del Cauca	2021	Dyspnea, cough	PCV2d
OP535528	Valle del Cauca	2021	Wasting /Dyspnea, cough	PCV2d
OP535529	Valle del Cauca	2021	Wasting	PCV2d
OP535530	Valle del Cauca	2021	Dyspnea, cough	PCV2a
OP535531	Valle del Cauca	2021	Dyspnea, cough	PCV2d

Table S3. Comparison of amino acid sequences between PCV2a-Cap and PCV2d-Cap of the Colombian strains in this study and reference strains available in the GenBank database.

	PCV2a		PCV2d	
Position	Isolates in our study (n=2)	Reference strains* (n=11)	Isolates in our study (n=57)	Reference strains (n=14)
8	F	6F/5Y	1Y/54F	F
21	Q	10Q/1L	Q	Q
46	T	1T/10N	T	T
47	A	5A/6T	T	T
51	R	10R/1C	R	R
53	F	F	I	I
59	A	8A/3R	K	K
63	S	5S/3R/3T	R	R
68	S	5S/6A	N	N
72	L	5L/6M	M	M
75	N	8N/3K	N	N
76	L	7L/3I	I	I
77	D	10D/1N	N	N
80	V	V	L	L
86	T	T	S	S
88	K	K	P	P
89	I	I	L	L
90	S	S	T	T
91	I	I	V	V
121	S	5S/6T	T	T
123	I	5I/6V	V	V
131	M	5M/3T/3P	T	T
133	V	5V/6A	55A/2S	A
134	P	6P/5T	N	N
136	Q	6Q/5L	L	L
137	S	1S/10T	T	T
151	P	P	T	T
169	S	10S/1A	36G/19R	G/R
185	M	6M/5L	L	L
190	S	S	T	T
191	1R/1K	9R/1G/1A	G	G
206	K	8K/2I/1T	I	I
207	H	1H/10Y	Y	Y
215	V	V	I	I
232	K	9K /2N	N	N
234	-	-	K	K

PCV2a reference sequences: AF055392, AF264042, DQ397521, HQ 202949, MK604484, KY388466, OL377291, OM677715, OL377678, HM038034, KX828215. PCV2d reference sequence: MZ558546. Dashes indicate amino acid residues at this position are absent among these PCV2 genotypes.

Table S4. Scores of histopathological lesions in collected tissues (lung, lymph node, spleen, and tonsil) from pigs with qPCR - PCV2 positive and with symptoms of PCVAD.

		Score		
**Organ**	**Microscopic lesion**	**Low (%)**	**Moderate (%)**	**Severe (%)**
Lung	Bronchitis/Bronchiolitis	4/60 (6.6)	7/60 (11.6)	0/60 (0)
	Thickening of alveolar septa	15/60 (25)	34/60 (56.6)	5/60 (8.3)
	Exudation in airways	8/60 (13.3)	6/60 (10)	20/60 (33.3)
	BALT Hyperplasia	11/60(18.3)	14/60 (23.3)	4/60 (6)
	Peribronchial/peribronchiolar/perivascular infiltration	15/60 (25)	20/60 (33.2)	3/60 (5)
	Pleuritis	8/60 (13.3)	3/60 (5)	2/60 (3.3)
lymph-nodes	Follicle lymphoid depletion	19/60 (31.6)	17/60 (28.3)	3/60 (5)
	Histiocytic infiltration	8/60 (13.3)	6/60 (10)	3/60 (5)
	Neutrophils in sinus system	17/60 (28.3)	11/60 (18.3)	10/60 (16.6)
	Intracytoplasmic inclusion bodies	1/60 (1.6)	0/60 (0)	1/60 (1.6)
Spleen	Mixed lymphoid depletion	17/56 (30.3)	17/56 (30.3)	7/56 (12.5)
	Histiocytic infiltration	5/56 (8.9)	8/56 (14.2)	0/56 (0)
	Presence of neutrophils	14/56 (25)	13/56 (23.2)	3/56 (5.3)
	Presence of multinucleate giants' cells	1/56 (1.7)	0/56 (0)	0/56 (0)
	Intracytoplasmic inclusion bodies	1/56 (1.7)	0/56 (0)	0/56 (0)
Tonsils	Indistinct follicles	0/44 (0)	1/44 (2.2)	0/44 (0)
	Lymphoid depletion	26/44 (59)	7/44 (15.9)	2/44 (4.5)
	Histiocytic infiltration	4/44 (9)	2/44 (4.5)	2/44 (4.5)
	Intracytoplasmic inclusion bodies	0/44 (0)	2/44 (4.5)	2.44 (4.5)

Table S5. EpiCC analysis and T cell epitope coverage of the PCV2-ORF-2 of the 57 sequences retrieved from Colombia.

						EpiCC score				T cell epitope Coverage %				Deficit relative to VacAB			VacAB % coverage increase over each monovalent vaccine		
Genbank fasta name from Table S2	Genbank accession Foss et al., 2023	WO	CircoMatch report - 2022	WO or CircoMatch id	EpiCC baseline	VacAB	VacAlt1	VacAlt2	VacAlt3	VacAB	VacAlt1	VacAlt2	VacAlt3	VacAlt1	VacAlt2	VacAlt3	VacAlt1	VacAlt2	VacAlt3
Atlantico1.3	OL377428	10		1.3 Atlantico Col 1d	10,349	8,397	6,282	6,610	6,940	81,14	60,70	63,87	67,06	20,44	17,27	14,08	33,67	27,03	20,99
Antioquia2.14	OL377436	10		2.14 Antioquia Col 9d	10,349	8,397	6,282	6,610	6,940	81,14	60,70	63,87	67,06	20,44	17,27	14,08	33,67	27,03	20,99
Antioquia2.15	OL377437	10		2.15 Antioquia Col 10d	10,349	8,397	6,282	6,610	6,940	81,14	60,70	63,87	67,06	20,44	17,27	14,08	33,67	27,03	20,99
Antioquia2.17			37	2,17	10,349	8,397	6,282	6,610	6,940	81,14	60,70	63,87	67,06	20,44	17,27	14,08	33,67	27,03	20,99
Antioquia2.21	OL377438	10		2.21 Antioquia Col 11d	10,349	8,397	6,282	6,610	6,940	81,14	60,70	63,87	67,06	20,44	17,27	14,08	33,67	27,03	20,99
Antioquia2.26			38	2,26	10,349	8,397	6,282	6,610	6,940	81,14	60,70	63,87	67,06	20,44	17,27	14,08	33,67	27,03	20,99
Antioquia2.27	OL377439	10		2.27 Antioquia Col 12d	10,349	8,397	6,282	6,610	6,940	81,14	60,70	63,87	67,06	20,44	17,27	14,08	33,67	27,03	20,99
Antioquia2.28			39	2,28	10,349	8,397	6,282	6,610	6,940	81,14	60,70	63,87	67,06	20,44	17,27	14,08	33,67	27,03	20,99
Antioquia2.29	OL377440	10		2.29 Antioquia Col 13d	10,349	8,397	6,282	6,610	6,940	81,14	60,70	63,87	67,06	20,44	17,27	14,08	33,67	27,03	20,99
Antioquia2.31			40	2,31	10,349	8,397	6,282	6,610	6,940	81,14	60,70	63,87	67,06	20,44	17,27	14,08	33,67	27,03	20,99
Antioquia2.32			40	2,32	10,349	8,397	6,282	6,610	6,940	81,14	60,70	63,87	67,06	20,44	17,27	14,08	33,67	27,03	20,99
Antioquia2.33			41	2,33	10,349	8,397	6,282	6,610	6,940	81,14	60,70	63,87	67,06	20,44	17,27	14,08	33,67	27,03	20,99
Antioquia2.34			41	2,34	10,425	8,397	6,281	6,609	6,940	80,55	60,25	63,40	66,57	20,30	17,15	13,98	33,69	27,05	20,99
Antioquia2.36			42	2,36	10,349	8,397	6,282	6,610	6,940	81,14	60,70	63,87	67,06	20,44	17,27	14,08	33,67	27,03	20,99
Antioquia2.38			43	2,38	10,349	8,397	6,282	6,610	6,940	81,14	60,70	63,87	67,06	20,44	17,27	14,08	33,67	27,03	20,99
Antioquia2.39			44	2,39	10,349	8,397	6,282	6,610	6,940	81,14	60,70	63,87	67,06	20,44	17,27	14,08	33,67	27,03	20,99
Antioquia2.42			45	2,42	10,349	8,397	6,282	6,610	6,940	81,14	60,70	63,87	67,06	20,44	17,27	14,08	33,67	27,03	20,99
Antioquia2.44	OL377441	10		2.44 Antioquia Col 14d	10,349	8,397	6,282	6,610	6,940	81,14	60,70	63,87	67,06	20,44	17,27	14,08	33,67	27,03	20,99
Antioquia2.45	OL377442	10		2.45 Antioquia Col 15d	10,349	8,397	6,282	6,610	6,940	81,14	60,70	63,87	67,06	20,44	17,27	14,08	33,67	27,03	20,99
Antioquia2.46	OL377443	10		2.46 Antioquia Col 16d	10,349	8,397	6,282	6,610	6,940	81,14	60,70	63,87	67,06	20,44	17,27	14,08	33,67	27,03	20,99
Antioquia2.47			46	2,47	10,349	8,397	6,282	6,610	6,940	81,14	60,70	63,87	67,06	20,44	17,27	14,08	33,67	27,03	20,99
Antioquia2.48			47	2,48	10,349	8,397	6,282	6,610	6,940	81,14	60,70	63,87	67,06	20,44	17,27	14,08	33,67	27,03	20,99
Antioquia2.49	OL377444	10		2.49 Antioquia Col 17d	10,349	8,397	6,282	6,610	6,940	81,14	60,70	63,87	67,06	20,44	17,27	14,08	33,67	27,03	20,99
Antioquia2.59			48	2,59	10,349	8,397	6,282	6,610	6,940	81,14	60,70	63,87	67,06	20,44	17,27	14,08	33,67	27,03	20,99
Cundinamarca3.1	OL377432	10		3.1 Cundinamarca Col 5d	10,389	8,397	6,282	6,610	6,940	80,83	60,47	63,62	66,80	20,36	17,20	14,02	33,67	27,03	20,99
Cundinamarca3.2	OL377433	10		3.2 Cundinamarca Col 6d	10,349	8,397	6,282	6,610	6,940	81,14	60,70	63,87	67,06	20,44	17,27	14,08	33,67	27,03	20,99
Cundinamarca3.3	OL377434	10		3.3 Cundinamarca Col 7d	10,389	8,397	6,282	6,610	6,940	80,83	60,47	63,62	66,80	20,36	17,20	14,02	33,67	27,03	20,99
Cundinamarca3.4			56	3,4	10,349	8,397	6,282	6,610	6,940	81,14	60,70	63,87	67,06	20,44	17,27	14,08	33,67	27,03	20,99
Cundinamarca3.7	OL377435	10		3.7 Cundinamarca Col 8d	10,501	8,741	6,625	6,953	7,284	83,24	63,09	66,21	69,36	20,15	17,03	13,87	31,94	25,72	20,00
Cundinamarca3.8	OL377672	11		Colombia_3_8d	10,425	8,397	6,281	6,609	6,940	80,55	60,25	63,40	66,57	20,30	17,15	13,98	33,69	27,05	20,99
Cundinamarca3.13			50	3,13	10,425	8,397	6,281	6,609	6,940	80,55	60,25	63,40	66,57	20,30	17,15	13,98	33,69	27,05	20,99

Cundinamarca3.16			51	3,16	10,425	8,397	6,281	6,609	6,940	80,55	60,25	63,40	66,57	20,30	17,15	13,98	33,69	27,05	20,99
Cundinamarca3.17			52	3,17	10,588	8,397	6,281	6,609	6,940	79,31	59,32	62,42	65,55	19,98	16,89	13,76	33,69	27,05	20,99
Cundinamarca3.18			52	3,18	10,588	8,397	6,281	6,609	6,940	79,31	59,32	62,42	65,55	19,98	16,89	13,76	33,69	27,05	20,99
Cundinamarca3.19			53	3,19	10,425	8,397	6,281	6,609	6,940	80,55	60,25	63,40	66,57	20,30	17,15	13,98	33,69	27,05	20,99
Cundinamarca3.20			53	3.20	10,425	8,397	6,281	6,609	6,940	80,55	60,25	63,40	66,57	20,30	17,15	13,98	33,69	27,05	20,99
Cundinamarca3.21			54	3,21	10,425	8,397	6,281	6,609	6,940	80,55	60,25	63,40	66,57	20,30	17,15	13,98	33,69	27,05	20,99
Cundinamarca3.22	OL377673	11		Colombia_3_22d	10,425	8,397	6,281	6,609	6,940	80,55	60,25	63,40	66,57	20,30	17,15	13,98	33,69	27,05	20,99
Cundinamarca3.23			55	3,23	10,425	8,397	6,281	6,609	6,940	80,55	60,25	63,40	66,57	20,30	17,15	13,98	33,69	27,05	20,99
Cundinamarca3.24			55	3,24	10,425	8,397	6,281	6,609	6,940	80,55	60,25	63,40	66,57	20,30	17,15	13,98	33,69	27,05	20,99
Meta			49	20L	10,425	8,397	6,281	6,609	6,940	80,55	60,25	63,40	66,57	20,30	17,15	13,98	33,69	27,05	20,99
Risaralda4.5			57	4,5	10,425	8,397	6,281	6,609	6,940	80,55	60,25	63,40	66,57	20,30	17,15	13,98	33,69	27,05	20,99
Risaralda4.6			57	4,6	10,425	8,397	6,281	6,609	6,940	80,55	60,25	63,40	66,57	20,30	17,15	13,98	33,69	27,05	20,99
Quindio4.7			58	4,7	10,425	8,397	6,281	6,609	6,940	80,55	60,25	63,40	66,57	20,30	17,15	13,98	33,69	27,05	20,99
Quindio4.8			58	4,8	10,425	8,397	6,281	6,609	6,940	80,55	60,25	63,40	66,57	20,30	17,15	13,98	33,69	27,05	20,99
Caldas4.9			59	4,9	10,425	8,397	6,281	6,609	6,940	80,55	60,25	63,40	66,57	20,30	17,15	13,98	33,69	27,05	20,99
Valle5.3	OL377429	10		5.3 Valle Col 2d	10,349	8,397	6,282	6,610	6,940	81,14	60,70	63,87	67,06	20,44	17,27	14,08	33,67	27,03	20,99
Valle5.4			66	5,4	10,349	8,397	6,282	6,610	6,940	81,14	60,70	63,87	67,06	20,44	17,27	14,08	33,67	27,03	20,99
Valle5.5	OL377430	10		5.5 Valle Col 3d	10,349	8,397	6,282	6,610	6,940	81,14	60,70	63,87	67,06	20,44	17,27	14,08	33,67	27,03	20,99
Valle5.6			67	5,6	10,240	7,802	7,132	7,378	6,434	76,19	69,65	72,05	62,83	6,54	4,14	13,36	9,39	5,75	21,26
Cauca5.11	OL377431	10		5.11 Valle Col 4d	10,349	8,397	6,282	6,610	6,940	81,14	60,70	63,87	67,06	20,44	17,27	14,08	33,67	27,03	20,99
Cauca5.12			60	5,12	10,349	8,397	6,282	6,610	6,940	81,14	60,70	63,87	67,06	20,44	17,27	14,08	33,67	27,03	20,99
Valle5.13			61	5,13	10,349	8,397	6,282	6,610	6,940	81,14	60,70	63,87	67,06	20,44	17,27	14,08	33,67	27,03	20,99
Valle5.20			62	5.20	10,349	8,397	6,282	6,610	6,940	81,14	60,70	63,87	67,06	20,44	17,27	14,08	33,67	27,03	20,99
Valle5.22			63	5,22	10,349	8,397	6,282	6,610	6,940	81,14	60,70	63,87	67,06	20,44	17,27	14,08	33,67	27,03	20,99
Valle5.29			64	5,29	10,155	6,765	6,349	6,595	6,432	66,62	62,52	64,94	63,34	4,10	1,67	3,28	6,55	2,58	5,18
Valle5.32			65	5,32	10,349	8,397	6,282	6,610	6,940	81,14	60,70	63,87	67,06	20,44	17,27	14,08	33,67	27,03	20,99

Table S6. EpiCC scores and T cell epitope coverage obtained on three vaccines under investigation and on 57 PCV2-ORF2 sequences classified according to each geographic province scrutinized.

		EpiCC Scores												T cell epitope coverage											
		Fostera (VacAB)			Porcilis (VacAlt1)			CircoFLEX (VacAlt2)			Mhyosphere (VacAlt3)			Fostera (VacAB)			Porcilis (VacAlt1)			CircoFLEX (VacAlt2)			Mhyosphere (VacAlt3)		
**Region**	**n**	**average**	**min**	**max**	**average**	**min**	**max**	**average**	**min**	**max**	**average**	**min**	**max**	**average**	**min**	**max**	**average**	**min**	**max**	**average**	**min**	**max**	**average**	**min**	**max**
All	57	8,364	6,765	8,741	6,304	6,281	7,132	6,629	6,610	7,378	6,928	6,940	7,284	80,59	66,62	83,24	60,75	59,32	69,65	63,88	62,42	72,05	66,76	62,83	69,36
Atlántico	1	8,397	8,397	8,397	6,282	6,282	6,282	6,610	6,610	6,610	6,940	6,940	6,940	81,14	81,14	81,14	60,70	60,70	60,70	63,87	63,87	63,87	67,06	67,06	67,06
Antioquia	23	8,397	8,397	8,397	6,282	6,281	6,282	6,610	6,609	6,610	6,940	6,940	6,940	81,11	80,55	81,14	60,68	60,25	60,70	63,85	63,40	63,87	67,04	66,57	67,06
Cundinamarca	17	8,419	8,397	8,741	6,303	6,281	6,625	6,631	6,609	6,953	6,962	6,940	7,284	80,67	79,31	83,24	60,39	59,32	63,09	63,54	62,42	66,21	66,71	65,55	69,36
Eje Cafetero	5	8,397	8,397	8,397	6,281	6,281	6,281	6,609	6,609	6,609	6,940	6,940	6,940	80,55	80,55	80,55	60,25	60,25	60,25	63,40	63,40	63,40	66,57	66,57	66,57
Valle del Cauca	11	8,195	6,765	8,397	6,365	6,282	7,132	6,678	6,595	7,378	6,848	6,432	6,940	79,37	66,62	81,14	61,68	60,70	69,65	64,71	63,87	72,05	66,34	62,83	67,06

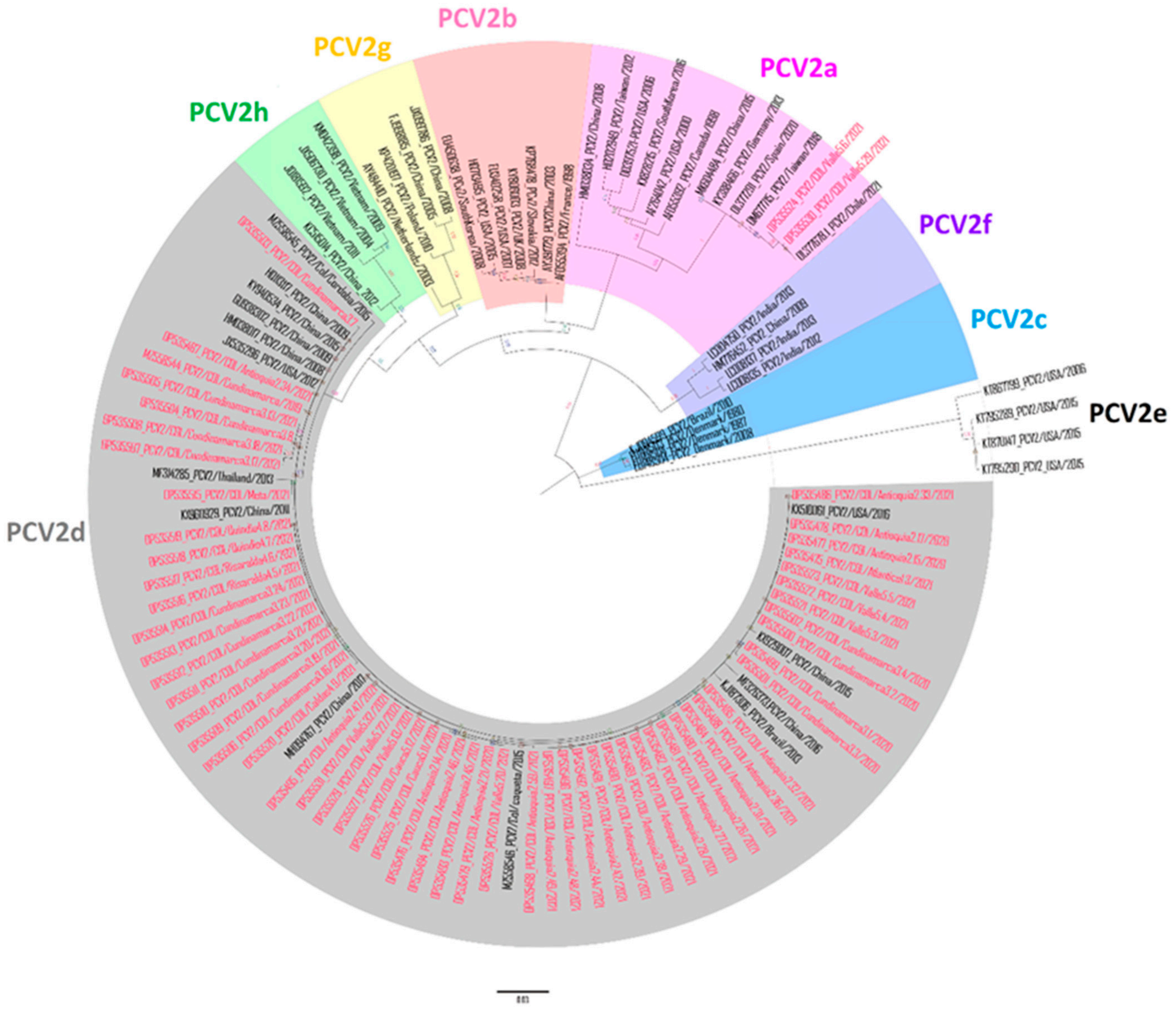

Figure S1. Maximum likelihood phylogenetic tree of 57 PCV2-ORF2 sequences identified in Colombia, inferred based on the alignment of the nucleotide sequences. The tree was constructed by ML analysis using the Tamura 3 parameter with gamma distribution, and tree topology was evaluated with 1000 bootstrap replicates. Colombian sequences generated in this study are highlighted in red and were juxtaposed with 53 sequences available in the NCBI GenBank nucleotide database.
